# Synthesis and Biological Evaluation of Benzimidazole Phenylhydrazone Derivatives as Antifungal Agents against Phytopathogenic Fungi

**DOI:** 10.3390/molecules21111574

**Published:** 2016-11-22

**Authors:** Xing Wang, Yong-Fei Chen, Wei Yan, Ling-Ling Cao, Yong-Hao Ye

**Affiliations:** 1 State & Local Joint Engineering Research Center of Green Pesticide Invention and Application, College of Plant Protection, Nanjing Agricultural University, Nanjing 210095, China; 2014102127@njau.edu.cn (X.W.); 2013102122@njau.edu.cn (Y.-F.C.); yanwei@njau.edu.cn (W.Y.); 2015202050@njau.edu.cn (L.-L.C.); 2 Key Laboratory of Integrated Management of Crop Diseases and Pests, Ministry of Education, Nanjing 210095, China

**Keywords:** benzimidazole, phenylhydrazone, antifungal activity

## Abstract

A series of benzimidazole phenylhydrazone derivatives (**6a**–**6ai**) were synthesized and characterized by ^1^H-NMR, ESI-MS, and elemental analysis. The structure of **6b** was further confirmed by single crystal X-ray diffraction as (*E*)-configuration. All the compounds were screened for antifungal activity against *Rhizoctonia solani* and *Magnaporthe oryzae* employing a mycelium growth rate method. Compound **6f** exhibited significant inhibitory activity against *R. solani* and *M. oryzae* with the EC_50_ values of 1.20 and 1.85 μg/mL, respectively. In vivo testing demonstrated that **6f** could effectively control the development of rice sheath blight (RSB) and rice blast (RB) caused by the above two phytopathogens. This work indicated that the compound **6f** with a benzimidazole phenylhydrazone scaffold could be considered as a leading structure for the development of novel fungicides.

## 1. Introduction

**Figure 1:**
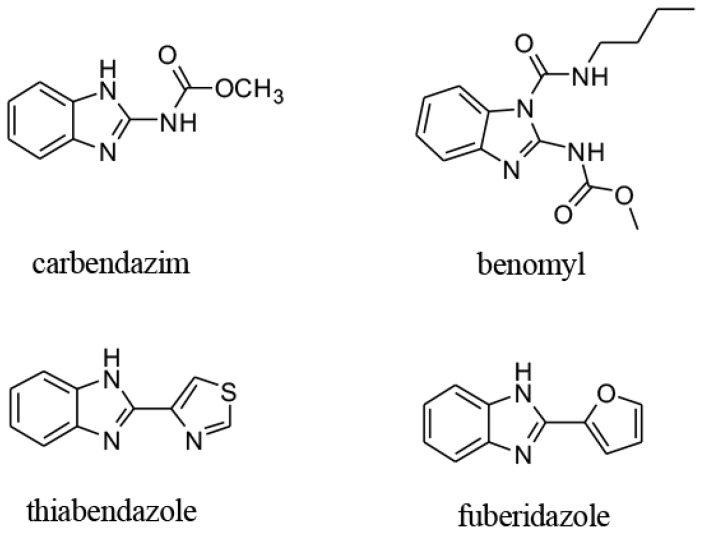
The commercialization of pesticide.

As an important chemical scaffold in medicinal chemistry and agrochemicals [1], benzimidazole derivatives have been attracting the interest of organic chemists for their varieties of biological activities, such as antifungal [2], anticancer [3], antibacterial [4], antioxidant [5], anti-inflammatory [6], and anti-parasitic [7]. In particular, benzimidazole fungicides, with benzimidazole as the core substructure (Figure 1), have been widely used throughout the world to fight against destructive plant pathogens, such as *Rhizoctonia solani*, *Botrytis cinerea*, *Fusarium graminearum*, and *Magnaporthe oryzae* [8,9]. However, excessive use of benzimidazole fungicides has led to a series of negative problems, such as pesticide residues, drug resistance and serious environmental pollution [10,11]. Therefore, the development of new benzimidazole fungicides with high activity, good selectivity and eco-friendly properties is extremely urgent.

In our previous work, molecules with phenylhydrazone showed significant antifungal and antioxidant activities [12,13]. Typically, 1,2,3-triazole phenylhydrazone derivatives displayed potent antifungal activity in vitro and in vivo. Inspired by these studies, we initiated a study to synthesize benzimidazole phenylhydrazone derivatives to screen high activity antifungal compounds. Their antifungal activities against two phytopathogenic fungi (*R. solani* and *M. oryzae*) were evaluated in vitro and in vivo, and their structure-antifungal activity relationships were also discussed.

## 2. Results and Discussion

### 2.1. Synthesis of Compounds

**Scheme 1:**
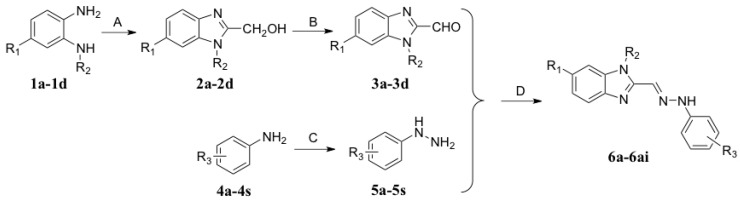
The synthetic routes to **6a**–**6ai**. Reagents and conditions: (**A**) (i) glycolic acid, HCl (20% aqueous), 100 °C, 5 h; (ii) NaOH (15% aqueous), pH 9; (**B**) MnO_2_, EtOAc, 65 °C, 1 h; (**C**) (i) NaNO_2_, HCl, 0 °C, 1 h; (ii) SnCl_2_, 0 °C; r.t., 2 h; (iii) NaOH (40% aqueous), pH 7; (**D**) MeOH, r.t., 2 h.

A total of 35 target compounds were synthesized following the routes outlined in Scheme 1. The compounds **2a**–**2d** were synthesized by the reaction of *o*-phenylenediamine with glycolic acid in 75%–85% yield. Then the compounds **2a**–**2d** were further oxidized to aldehydes (**3a**–**3d**) using MnO_2_ in ethyl acetate. The aldehydes were purified by filtration to remove the excess MnO_2_, then compounds **3a**–**3d** were obtained as white crystals in 60%–76% yield. The anilines **4a**–**4s** were diazotized by NaNO_2_, then restored by SnCl_2_ to obtain substituted phenylhydrazines **5a**–**5s** in 55%–80% yield. Finally, **3a**–**3d** and **5a**–**5s** were condensed to form the -C=N-NH- bond, giving benzimidazole derivatives **6a**–**6ai** in 45%–80% yield. ^1^H-NMR, MS (ESI), and elemental analysis (CHN) data of the target compounds were in fully accordance with their assigned structures. Among them, compounds **6a**, **6q**, **6t**, **6x**, and **6z** had been reported previously [14,15], while the remaining 30 compounds were first reported in this study.

### 2.2. Crystal Structure of Compound ***6b***

**Figure 2:**
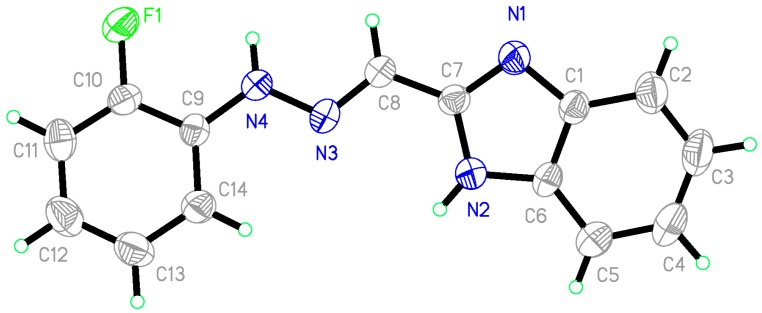
X-ray diffraction structure of **6b**, thermal ellipsoids was drawn on the 35% probability.

Among these compounds, the crystal structure of compound **6b** was determined by X-ray diffraction analysis. Figure 2 gave a perspective view of **6b** with the atomic labelling system. The X-ray data had been deposited at the Cambridge Crystallographic Data Centre with the CCDC number 1038194. The result demonstrated that the -C=N-NH- bond bears an (*E*)-configuration rather than (*Z*)-configuration.

### 2.3. Antifungal Activities In Vitro

Different concentrations of compounds **6a**–**6ai** were evaluated for their antifungal activities in vitro against *R. solani* and *M. oryzae*. The EC_50_ values were calculated using linear-regression analysis, with validamycin A, carbendazim and isoprothiolane as positive controls (Table 1).

Among 35 compounds, 27 compounds showed significant inhibitory activities against *R. solani*. **6l**–**6m**, **6q**–**6t** were slightly weaker than validamycin A (5.07 μg/mL), while the other 20 compounds were more potent than validamycin A. Interestingly, seven compounds were superior to carbendazim (1.84 μg/mL). Taking single halogen substituents (**6k**–**6m**) into account, it was obvious that **6m** (2,4-2Cl, 2.83 μg/mL) > **6l** (2,6-2Cl, 5.85 μg/mL) > **6k** (2,5-2Cl, 7.47 μg/mL) (“>” means more potent). The compound **6ah** (0.93 μg/mL) showed the strongest activity, which was much more superior to that of carbendazim (1.84 μg/mL). When R_2_ was methyl, the activity of compounds (**6a** and **6t**, **6c** and **6u**) was significantly decreased; H was compared to R_2_.

Some target compounds showed great inhibitory activities against *M. oryzae*. Among these 35 compounds, **6f** showed potent inhibiting effects with EC_50_ of 1.85 μg/mL, compared to carbendazim (EC_50_ of 1.87 μg/mL). Taking single halogen substituents (**6b**–**6d**) and (**6h**–**6j**) into account, it was obvious that **6b** (2F, 6.73 μg/mL) > **6d** (4F, 8.29 μg/mL) > **6c** (3F, 9.08 μg/mL), **6h** (2Cl, 5.47 μg/mL) > **6j** (4Cl, 8.71, μg/mL) > **6i** (3Cl, 13.66 μg/mL).

Based on the results of the antifungal activities shown in Table 1, **6f** was selected for further antifungal activity tests in vivo.

### 2.4. Inhibition of ***6f*** on the Sclerotia Germination of R. solani

As shown in Table 2, when grain dry sclerotia was inoculated, germination could be observed in the water of the blank control after a certain incubation period (about four days) at 25 °C in the dark. **6f** had better controlling effects than sclerotia.

### 2.5. Protective Activity of ***6f*** against Rice Sheath Blight (RSB) In Vivo

**Figure 3:**
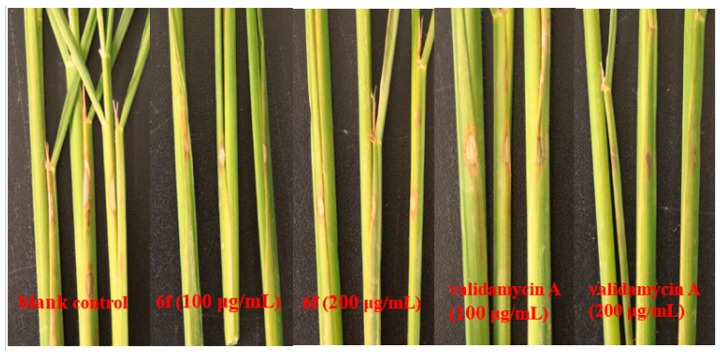
Protective activity of **6f** against rice sheath blight (RSB).

Compound **6f** was selected for evaluating the protective activity against RSB caused by *R. solani*. As shown in Figure 3 and Table 3, four days after inoculating with *R. solani*, significant differences could be observed between the treated and untreated groups. Tan spots appeared on the leaf sheath of the blank control, and the scab length reached 16.4 mm. When at a concentration of 200 μg/mL, the protective effect of **6f** could reach 68.9% in vivo, close to validamycin A (73.8%), which was commonly used as a fungicide against RSB. When at a concentration of 100 μg/mL, the protective effect of **6f** and validamycin A reached 66.5% and 61.0% respectively. Thus, **6f** showed similar activity with validamycin A against RSB in vivo.

### 2.6. Inhibition of ***6f*** on the Conidium Germination of M. oryzae

**Figure 4:**
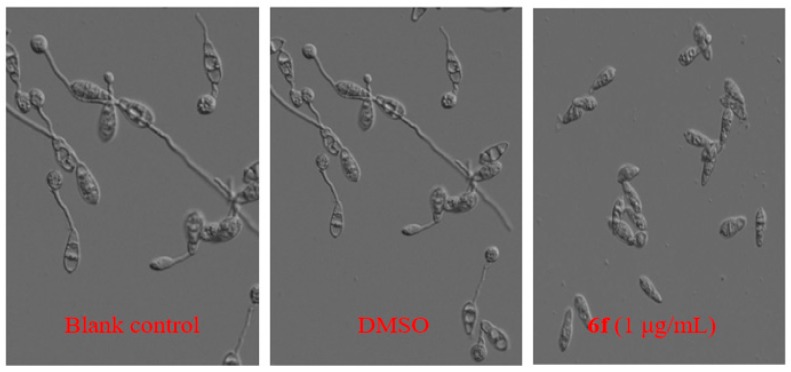
The effect of **6f** on the conidium germination rate of *M. oryzae*.

As shown in Figure 4, conidia of *M. oryzae* were inoculated after 24 h, germination could be observed in the water of the blank control under the microscope with a germination rate of 100%, and 1% DMSO had no effect on the conidium germination. The conidia could not form the germ tube or appressorium when incubated with **6f** at the concentration of 1.0 μg/mL (1% DMSO).

### 2.7. Protective Activity of ***6f*** against Rice Blast (RB) In Vivo

**Figure 5:**
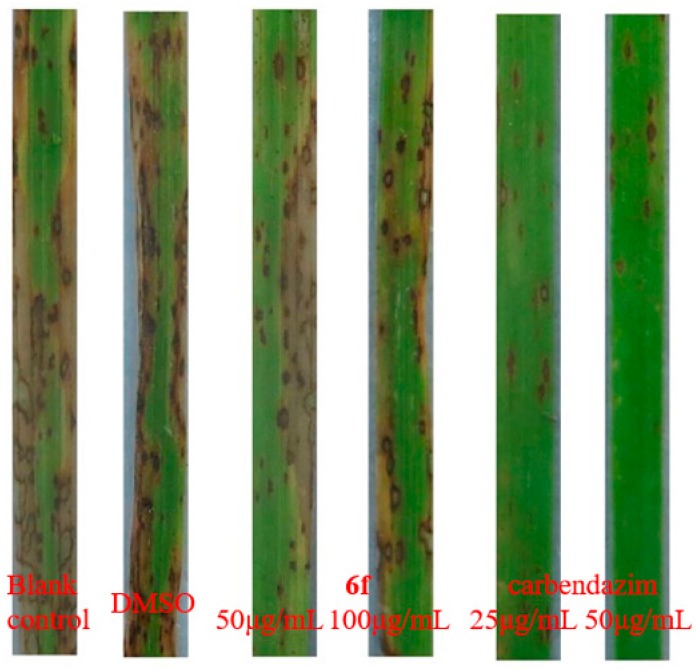
Protective activity of **6f** against rice blast (RB).

The efficacy of the protective activity experiment is shown in Figure 5 and Table 4, fungus spores were inoculated on leaves, blast lesions could be observed after 7 days. At a concentration of 50 μg/mL, the protective effect of **6f** could reach 21.6%, whereas carbendazim treatment resulted in 88.0%.

## 3. Materials and Methods

### 3.1. Reagents and Analysis

All reagents bought from Alfa Aesar (Ward Hill, MA, USA), Aladdin (Shanghai, China) or Sinopharm Chemical Reagent Co., Ltd. (Beijing, China) were pure analytical grades and used without further treatments. Reactions were monitored by TLC using silica gel coated glass slides (silica-gel 60 GF254, Qingdao Haiyang Chemical, Qingdao, China). Melting points were measured on WRS-1B digital melting-point apparatus (SPSIC, Shanghai, China), uncorrected. ^1^H-NMR spectra were recorded on a Bruker Avance III 400 NMR spectrometer (Bruker, Stuttgart, Germany). The chemical shifts (δ) are reported in ppm with reference to internal TMS, and coupling constants (*J*) are given in Hz. ESI-MS spectra were recorded on a Bruker UHR-TOF maxis (Bruker, Billerica, MA, USA). X-ray single crystal diffraction analysis was conducted on a Bruker D8 Venture diffractometer (Bruker, Karlsruhe, German). Elemental analyses were performed on a Elementar Vario MICRO instrument (Elementar, Langenselbold, German) and were within ±0.4% of the theoretical values.

### 3.2. Synthesis

#### 3.2.1. Synthesis and Purification of Compound **2a**–**2d**

The procedures were conducted according to the literature [16]. Glycolic acid (15.24 g, 0.2 mol) was dissolved in 40 mL HCl (20% aqueous) in the flask. When the temperature reached 65 °C, compound **1a**–**1d** (16.27 g, 0.15 mol) was added at three times, then refluxed at 100 °C for 5 h. After cooling to room temperature, the solution was basified with NaOH (15%, aqueous) until it reached pH 9.0 to precipitate the desired compounds **2a**–**2d** (in 75%–85% yield).

#### 3.2.2. Synthesis and Purification of Compound **3a**–**3d**

A solution of **2a**–**2d** (2 mmol), MnO_2_ (3.48 g, 40 mmol) was added to EtOAc (120 mL), then refluxed at 65 °C for 1 h (monitored by TLC). Afterwards the solution was filtered, and concentrated in vacuo to give pure compounds **3a**–**3d** (in 60%–76% yield) [17].

#### 3.2.3. Synthesis and Purification of Compound **5a**–**5s**

Substituted aniline **4a**–**4s** (50 mmol) was dissolved in the 50 mL HCl (18%, aqueous) in the ice bath. NaNO_2_ (50 mmol) dissolved in 50 mL water was added dropwise. The reaction mixture was stirred for 1 h to obtain a clear solution. Then the solution of SnCl_2_ (0.1 mol) in 30 mL of concentrated HCl was added dropwise at 0 °C. The mixture was stirred at room temperature for 2 h. Afterwards, the mixture was extracted with 50 mL EtOAc and the organic impurities were discarded. Then the solution was basified with NaOH (40%, aqueous) until it reached pH 7.0. The reaction mass was extracted with EtOAc three times. Finally, substituted phenylhydrzine **5a**–**5s** was afforded after being vapored under reduced pressure (in 55%–80% yield) [12].

#### 3.2.4. Synthesis and Purification of Compound **6a**–**6ai**

The equimolar aldehyde **3a**–**3d** (1 mmol) and substituted phenylhydrazine **5a**–**5s** (1 mmol) were mixed in CH_3_OH (10 mL) and stirred at room temperature [18]. After about 2 h, the reaction was completed (monitored by TLC). The residual crude was purified via silica gel column chromatogram using a gradient mixture of petroleum ether and ethyl acetate to obtain the pure target compounds **6a**–**6ai** (in 45–80% yield).

*(E)-2-((2-Phenylhydrazono)methyl)-1H-benzo[d]imidazole* (**6a**): Yellow power, yield 64.2%, m.p. 185.3–186.1 °C. ^1^H-NMR (400 MHz, DMSO-*d*_6_) δ 12.52 (s, 1H), 10.90 (s, 1H), 7.88 (s, 1H), 7.53 (s, 2H), 7.31–7.26 (m, 2H), 7.25 (t, *J* = 4.2 Hz, 2H), 7.18 (d, *J* = 2.8 Hz, 2H), 6.84 (dd, *J* = 9.7, 4.2 Hz, 1H). MS (ESI): 237.10 (C_14_H_13_N_4_, [M + H]^+^). Anal. Calcd. For C_14_H_12_N_4_: C, 71.17; H, 5.12; N, 23.71. Found: C, 70.95; H, 5.19; N, 23.49.

*(E)-2-((2-(2-Fluorophenyl)hydrazono)methyl)-1H-benzo[d]imidazole* (**6b**): Sandybrown power, yield 72.8%, 201.8–202.4 °C. ^1^H-NMR (400 MHz, DMSO-*d*_6_) δ 12.64 (s, 1H), 10.89 (s, 1H), 8.18 (s, 1H), 7.83 (t, *J* = 8.2 Hz, 1H), 7.62–7.49 (m, 2H), 7.25–7.14 (m, 4H), 6.86 (dd, *J* = 13.0, 7.0 Hz, 1H). MS (ESI): 255.10 (C_14_H_12_FN_4_, [M + H]^+^). Anal. Calcd. For C_14_H_11_FN_4_: C, 66.13; H, 4.36; N, 22.04. Found: C, 66.34; H, 4.11; N, 21.89.

*(E)-2-((2-(3-Fluorophenyl)hydrazono)methyl)-1H-benzo[d]imidazole* (**6c**): Yellow power, yield 69.3%, m.p. 203.5–205.9 °C. ^1^H-NMR (400 MHz, DMSO-*d*_6_) δ 12.61 (s, 1H), 11.09 (s, 1H), 7.90 (d, *J* = 0.6 Hz, 1H), 7.55 (s, 2H), 7.33–7.26 (m, 1H), 7.26–7.17 (m, 3H), 6.91 (dd, *J* = 8.1, 1.2 Hz, 1H), 6.63 (td, *J* = 8.3, 2.0 Hz, 1H). MS (ESI): 255.10 (C_14_H_12_FN_4_, [M + H]^+^). Anal. Calcd. For C_14_H_11_FN_4_: C, 66.13; H, 4.36; N, 22.04. Found: C, 66.34; H, 4.11; N, 21.89.

*(E)-2-((2-(4-Fluorophenyl)hydrazono)methyl)-1H-benzo[d]imidazole* (**6d**): Yellow power, yield 76.8%, m.p. 199.2–199.4 °C. ^1^H-NMR (400 MHz, DMSO-*d*_6_) δ 12.54 (s, 1H), 10.90 (s, 1H), 7.86 (s, 1H), 7.52 (s, 2H), 7.24 (dt, *J* = 10.2, 4.1 Hz, 2H), 7.21–7.11 (m, 4H). MS (ESI): 255.10 (C_14_H_12_FN_4_, [M + H]^+^). Anal. Calcd. For C_14_H_11_FN_4_: C, 66.13; H, 4.36; N, 22.04. Found: C, 66.34; H, 4.11; N, 21.89.

*(E)-2-((2-(4-(Trifluoromethoxy)phenyl)hydrazono)methyl)-1H-benzo[d]imidazole* (**6e**): White power, yield 54.1%, m.p. 207.3–207.9 °C. ^1^H-NMR (400 MHz, DMSO-*d*_6_) δ 12.61 (s, 1H), 11.07 (s, 1H), 7.91 (d, *J* = 0.6 Hz, 1H), 7.54 (s, 2H), 7.33–7.28 (m, 4H), 7.19 (d, *J* = 2.8 Hz, 2H). MS (ESI): 321.09 (C_15_H_12_F_3_N_4_O, [M + H]^+^). Anal. Calcd. For C_15_H_11_F_3_N_4_O: C, 56.25; H, 3.46; N, 17.49. Found: C, 56.27; H, 3.65; N, 17.07.

*(E)-2-((2-(2,4-Difluorophenyl)hydrazono)methyl)-1H-benzo[d]imidazole* (**6f**): Brown power, yield 65.5%, m.p. 200.2–200.3 °C. ^1^H-NMR (400 MHz, DMSO-*d*_6_) δ 12.68 (s, 1H), 11.05 (s, 1H), 8.18 (s, 1H), 7.70–7.64 (m, 1H), 7.61 (d, *J* = 7.8 Hz, 1H), 7.50 (d, *J* = 7.7 Hz, 1H), 7.29–7.21 (m, 2H), 7.18 (dd, *J* = 13.4, 5.6 Hz, 1H), 6.64 (ddd, *J* = 11.9, 8.5, 3.3 Hz, 1H). MS (ESI): 273.09 (C_14_H_11_F_2_N_4_, [M + H]^+^). Anal. Calcd. For C_14_H_10_F_2_N_4_: C, 61.76; H, 3.70; N, 20.58. Found: C, 61.89; H, 3.83; N, 20.49.

*(E)-2-((2-(2,5-Difluorophenyl)hydrazono)methyl)-1H-benzo[d]imidazole* (**6g**): Pale yellow power, yield 59.6%, m.p. 247.1–248.2 °C. ^1^H-NMR (400 MHz, DMSO-*d*_6_) δ 12.67 (s, 1H), 11.04 (s, 1H), 8.18 (s, 1H), 7.70–7.63 (m, 1H), 7.61 (d, *J* = 7.7 Hz, 1H), 7.50 (d, *J* = 7.7 Hz, 1H), 7.29–7.21 (m, 2H), 7.21–7.14 (m, 1H), 6.67–6.59 (m, 1H). MS (ESI): 273.09 (C_14_H_11_F_2_N_4_, [M + H]^+^). Anal. Calcd. For C_14_H_10_F_2_N_4_: C, 61.76; H, 3.70; N, 20.58. Found: C, 61.89; H, 3.83; N, 20.49.

*(E)-2-((2-(2-Chlorophenyl)hydrazono)methyl)-1H-benzo[d]imidazole* (**6h**): Brown power, yield 72.6%, m.p. 198.8–199.3 °C. ^1^H-NMR (400 MHz, DMSO-*d*_6_) δ 12.65 (s, 1H), 10.54 (s, 1H), 8.35 (s, 1H), 7.88 (d, *J* = 8.2 Hz, 1H), 7.56 (s, 2H), 7.38 (d, *J* = 7.9 Hz, 1H), 7.33 (t, *J* = 7.7 Hz, 1H), 7.21 (s, 2H), 6.88 (dd, *J* = 10.9, 4.3 Hz, 1H). MS (ESI): 271.05 (C_14_H_12_ClN_4_, [M + H]^+^). Anal. Calcd. For C_14_H_11_ClN_4_: C, 62.11; H, 4.10; N, 20.70. Found: C, 61.94; H, 4.19; N, 20.29.

*(E)-2-((2-(3-Chlorophenyl)hydrazono)methyl)-1H-benzo[d]imidazole* (**6i**): Pale yellow power, yield 66.7%, m.p. 212.0–212.2 °C. ^1^H-NMR (400 MHz, DMSO-*d*_6_) δ 12.62 (s, 1H), 11.06 (s, 1H), 7.89 (s, 1H), 7.59 (d, *J* = 7.9 Hz, 1H), 7.49 (d, *J* = 7.7 Hz, 1H), 7.43 (s, 1H), 7.28 (t, *J* = 8.0 Hz, 1H), 7.22 (t, *J* = 7.5 Hz, 1H), 7.16 (t, *J* = 7.5 Hz, 1H), 7.03 (d, *J* = 8.2 Hz, 1H), 6.86 (dd, *J* = 7.8, 1.2 Hz, 1H). MS (ESI): 271.05 (C_14_H_12_ClN_4_, [M + H]^+^). Anal. Calcd. For C_14_H_11_ClN_4_: C, 62.11; H, 4.10; N, 20.70. Found: C, 61.94; H, 4.19; N, 20.29.

*(E)-2-((2-(4-Chlorophenyl)hydrazono)methyl)-1H-benzo[d]imidazole* (**6j**): Yellow power, yield 72.3%, m.p. 194.4–194.5 °C. ^1^H-NMR (400 MHz, DMSO-*d*_6_) δ 12.59 (s, 1H), 11.02 (s, 1H), 7.88 (s, 1H), 7.59 (d, *J* = 7.8 Hz, 1H), 7.48 (d, *J* = 7.8 Hz, 1H), 7.34 (d, *J* = 2.0 Hz, 1H), 7.33–7.31 (m, 1H), 7.28–7.25 (m, 1H), 7.24 (d, *J* = 2.0 Hz, 1H), 7.24–7.19 (m, 1H), 7.16 (t, *J* = 7.6 Hz, 1H). MS (ESI): 271.05 (C_14_H_12_ClN_4_, [M + H]^+^). Anal. Calcd. For C_14_H_11_ClN_4_: C, 62.11; H, 4.10; N, 20.70. Found: C, 61.94; H, 4.19; N, 20.29.

*(E)-2-((2-(2,4-Dichlorophenyl)hydrazono)methyl)-1H-benzo[d]imidazole* (**6k**): Yellow power, yield 69.4%, m.p. 212.7–212.8 °C. ^1^H-NMR (400 MHz, DMSO-*d*_6_) δ 12.71 (s, 1H), 10.65 (s, 1H), 8.36 (d, *J* = 0.8 Hz, 1H), 7.89 (d, *J* = 8.9 Hz, 1H), 7.53 (d, *J* = 2.3 Hz, 3H), 7.41 (dd, *J* = 8.9, 2.3 Hz, 1H), 7.21 (s, 2H). MS (ESI): 305.02 (C_14_H_11_C_l2_N_4_, [M + H]^+^). Anal. Calcd. For C_14_H_10_Cl_2_N_4_: C, 55.10; H, 3.30; N, 18.36. Found: C, 55.58; H, 3.56; N, 17.88.

*(E)-2-((2-(2,5-Dichlorophenyl)hydrazono)methyl)-1H-benzo[d]imidazole* (**6l**): Yellow power, yield 60.1%, m.p. 215.2–215.3 °C. ^1^H-NMR (400 MHz, DMSO-*d*_6_) δ 12.87 (s, 1H), 10.70 (s, 1H), 8.38 (s, 1H), 7.94 (d, *J* = 2.5 Hz, 1H), 7.58 (dd, *J* = 5.6, 3.5 Hz, 2H), 7.40 (t, *J* = 6.1 Hz, 1H), 7.26–7.20 (m, 2H), 6.91 (dd, *J* = 8.5, 2.5 Hz, 1H). MS (ESI): 305.02 (C_14_H_11_C_l2_N_4_, [M + H]^+^). Anal. Calcd. For C_14_H_10_Cl_2_N_4_: C, 55.10; H, 3.30; N, 18.36. Found: C, 55.58; H, 3.56; N, 17.88.

*(E)-2-((2-(2,6-Dichlorophenyl)hydrazono)methyl)-1H-benzo[d]imidazole* (**6m**): Pale yellow power, yield 72.3%, m.p. 212.5–212.9 °C.^1^H-NMR (400 MHz, DMSO-*d*_6_) δ 12.38 (s, 1H), 10.13 (s, 1H), 7.87 (s, 1H), 7.56 (d, *J* = 8.1 Hz, 3H), 7.43 (s, 1H), 7.25 (t, *J* = 8.1 Hz, 1H), 7.16 (s, 2H). MS (ESI): 305.02 (C_14_H_11_C_l2_N_4_, [M + H]^+^). Anal. Calcd. For C_14_H_10_Cl_2_N_4_: C, 55.10; H, 3.30; N, 18.36. Found: C, 55.58; H, 3.56; N, 17.88.

*(E)-2-((2-(3,5-Dichlorophenyl)hydrazono)methyl)-1H-benzo[d]imidazole* (**6n**): Yellow power, yield 55.8%, m.p. 260.8–261.0 °C. ^1^H-NMR (400 MHz, DMSO-*d*_6_) δ 12.71 (s, 1H), 11.20 (s, 1H), 7.91 (s, 1H), 7.56 (s, 2H), 7.24 (d, *J* = 1.2 Hz, 2H), 7.21 (s, 2H), 6.97 (t, *J* = 1.8 Hz, 1H). MS (ESI): 305.02 (C_14_H_11_C_l2_N_4_, [M + H]^+^). Anal. Calcd. For C_14_H_10_Cl_2_N_4_: C, 55.10; H, 3.30; N, 18.36. Found: C, 55.58; H, 3.56; N, 17.88.

*(E)-2-((2-(2-Bromophenyl)hydrazono)methyl)-1H-benzo[d]imidazole* (**6o**): Brown power, yield 65.9%, m.p. 202.2–202.3 °C. ^1^H-NMR (400 MHz, DMSO-*d*_6_) δ 12.65 (s, 1H), 10.32 (s, 1H), 8.39 (s, 1H), 7.86 (dd, *J* = 8.3, 1.3 Hz, 1H), 7.64–7.50 (m, 3H), 7.39–7.34 (m, 1H), 7.19 (t, *J* = 12.5 Hz, 2H), 6.83 (td, *J* = 7.9, 1.5 Hz, 1H). MS (ESI): 315.02 (C_14_H_12_BrN_4_, [M + H]^+^). Anal. Calcd. For C_14_H_11_BrN_4_: C, 53.35; H, 3.52; N, 17.78. Found: C, 53.38; H, 3.67; N, 17.28.

*(E)-2-((2-(4-Bromophenyl)hydrazono)methyl)-1H-benzo[d]imidazole* (**6p**): Brown power, yield: 75.2%, m.p. 202.2–202.5 °C. ^1^H-NMR (400 MHz, DMSO-*d*_6_) δ 12.59 (s, 1H), 11.02 (s, 1H), 7.88 (s, 1H), 7.53 (s, 2H), 7.45 (d, *J* = 8.8 Hz, 2H), 7.23–7.16 (m, 4H). MS (ESI): 315.02 (C_14_H_12_BrN_4_, [M + H]^+^). Anal. Calcd. For C_14_H_11_BrN_4_: C, 53.35; H, 3.52; N, 17.78. Found: C, 53.38; H, 3.67; N, 17.28.

*(E)-2-((2-(4-Methoxyphenyl)hydrazono)methyl)-1H-benzo[d]imidazole* (**6q**): Brown power, yield: 69.5%, m.p. 196.8–196.9 °C. ^1^H-NMR (400 MHz, DMSO-*d_6_*) δ:12.46 (s, 1H), 10.74 (s, 1H), 7.81 (s, 1H), 7.51 (dd, *J* = 5.2, 3.6 Hz, 2H), 7.19 (d, *J* = 2.1 Hz, 1H), 7.17 (d, *J* = 3.2 Hz, 2H), 7.15 (d, *J* = 3.2 Hz, 1H), 6.91 (d, *J* = 2.1 Hz, 1H), 6.90–6.88 (m, 1H), 3.72 (s, 3H). MS (ESI): 267.10 (C_15_H_15_N_4_O, [M + H]^+^). Anal. Calcd. For C_15_H_14_FN_4_O: C, 67.65; H, 5.30; N, 21.04. Found: C, 67.38; H, 5.21; N, 21.29.

*(E)-4-(2-((1H-Benzo[d]imidazol-2-yl)methylene)hydrazinyl)benzonitrile* (**6r**): Brown power, yield 67.9%, m.p. 260.1–260.2 °C. ^1^H-NMR (400 MHz, DMSO-*d*_6_) δ 12.71 (s, 1H), 11.41 (s, 1H), 7.72 (d, *J* = 8.9 Hz, 2H), 7.53 (d, *J* = 35.5 Hz, 2H), 7.35 (d, *J* = 8.5 Hz, 2H), 7.28–7.15 (m, 2H). MS (ESI): 262.09 (C_15_H_12_N_5_, [M + H]^+^). Anal. Calcd. For C_15_H_11_N_5_: C, 68.95; H, 4.24; N, 26.80. Found: C, 68.74; H, 4.13; N, 26.99.

*(E)-2-((2-(3,4-Dimethylphenyl)hydrazono)methyl)-1H-benzo[d]imidazole* (**6s**): Brown power, yield 56.7%, m.p. 203.9–204.5 °C. ^1^H-NMR (400 MHz, DMSO-*d*_6_) δ 12.48 (s, 1H), 10.76 (s, 1H), 7.82 (s, 1H), 7.56–7.47 (m, 2H), 7.17 (dd, *J* = 5.8, 2.9 Hz, 2H), 7.08–7.00 (m, 2H), 6.99–6.94 (m, 1H), 2.22 (s, 3H), 2.16 (s, 3H). MS (ESI): 265.12 (C_16_H_17_N_4_, [M + H]^+^). Anal. Calcd. For C_16_H_16_N_4_: C, 72.70; H, 6.10; N, 21.20. Found: C, 72.24; H, 6.19; N, 21.09.

*(E)-1-Methyl-2-((2-phenylhydrazono)methyl)-1H-benzo[d]imidazole* (**6t**): Yellow power, yield: 65.2%, m.p. 201.5–201.7 °C. ^1^H-NMR (400 MHz, DMSO-*d*_6_) δ 10.91 (s, 1H), 8.04 (s, 1H), 7.65–7.55 (m, 2H), 7.34–7.25 (m, 3H), 7.21 (t, *J* = 7.1 Hz, 1H), 7.11 (d, *J* = 7.7 Hz, 2H), 6.85 (t, *J* = 7.3 Hz, 1H), 4.14 (s, 3H). MS (ESI): 251.10 (C_15_H_15_N_4_, [M + H]^+^). Anal. Calcd. For C_15_H_14_N_4_: C, 71.98; H, 5.64; N, 22.38. Found: C, 71.73; H, 5.31; N, 22.69.

*(E)-2-((2-(3-Fluorophenyl)hydrazono)methyl)-1-methyl-1H-benzo[d]imidazole* (**6u**): Yellow power, yield: 54.6%, m.p. 219.7–319.8 °C. ^1^H-NMR (400 MHz, DMSO-*d*_6_) δ 11.09 (s, 1H), 8.06 (s, 1H), 7.64–7.58 (m, 2H), 7.30 (dddd, *J* = 9.4, 7.2, 5.2, 2.7 Hz, 2H), 7.22 (ddd, *J* = 8.2, 7.2, 1.2 Hz, 1H), 6.91–6.84 (m, 2H), 6.68–6.61 (m, 1H), 4.13 (s, 3H). MS (ESI): 269.14 (C_15_H_14_FN_4_, [M + H]^+^). Anal. Calcd. For C_15_H_13_FN_4_: C, 67.15; H, 4.88; N, 20.88. Found: C, 67.23; H, 4.71; N, 20.69.

*(E)-2-((2-(2,4-Difluorophenyl)hydrazono)methyl)-1-methyl-1H-benzo[d]imidazole* (**6v**): Yellow power, yield: 64.1%, m.p. 191.6–191.8 °C. ^1^H-NMR (400 MHz, DMSO-*d*_6_) δ 10.81 (s, 1H), 8.31 (s, 1H), 7.61 (t, *J* = 8.4 Hz, 2H), 7.47 (td, *J* = 9.3, 5.9 Hz, 1H), 7.30 (ddd, *J* = 14.6, 8.2, 2.8 Hz, 2H), 7.23 (dd, *J* = 11.1, 4.0 Hz, 1H), 7.08 (t, *J* = 8.6 Hz, 1H), 4.12 (s, 3H). MS (ESI): 287.11 (C_15_H_13_F_2_N_4_, [M + H]^+^). Anal. Calcd. For C_15_H_12_F_2_N_4_: C, 62.93; H, 4.22; N, 19.57. Found: C, 62.83; H, 4.11; N, 19.66.

*(E)-2-((2-(2,5-Dichlorophenyl)hydrazono)methyl)-1-methyl-1H-benzo[d]imidazole* (**6w**): Yellow power, yield: 72.3%, m.p. 206.5–207.2 °C. ^1^H-NMR (400 MHz, DMSO-*d*_6_) δ 10.68 (s, 1H), 8.56 (s, 1H), 7.64 (dd, *J* = 16.3, 8.0 Hz, 2H), 7.44 (d, *J* = 8.2 Hz, 2H), 7.32 (t, *J* = 7.6 Hz, 1H), 7.24 (t, *J* = 7.5 Hz, 1H), 6.93 (dd, *J* = 8.5, 2.2 Hz, 1H), 4.13 (s, 3H). MS (ESI): 319.06 (C_15_H_13_Cl_2_N_4_, [M + H]^+^). Anal. Calcd. For C_15_H_12_Cl_2_N_4_: C, 56.44; H, 3.79; N, 17.55. Found: C, 56.31; H, 4.01; N, 17.61.

*(E)-2-((2-(4-Chlorophenyl)hydrazono)methyl)-1-methyl-1H-benzo[d]imidazole* (**6x**): Yellow power, yield: 67.5%, m.p. 213.2–213.5 °C. ^1^H-NMR (400 MHz, DMSO-*d*_6_) δ 11.02 (s, 1H), 8.05 (d, *J* = 1.1 Hz, 1H), 7.63–7.57 (m, 2H), 7.34 (d, *J* = 2.0 Hz, 1H), 7.33–7.30 (m, 1H), 7.29–7.26 (m, 1H), 7.22 (ddd, *J* = 8.2, 7.1, 1.3 Hz, 1H), 7.11 (d, *J* = 2.1 Hz, 1H), 7.09 (d, *J* = 2.1 Hz, 1H), 4.12 (s, 3H). MS (ESI): 285.09 (C_15_H_14_ClN_4_, [M + H]^+^). Anal. Calcd. For C_15_H_13_ClN_4_: C, 63.27; H, 4.60; N, 19.68. Found: C, 63.11; H, 4.69; N, 19.76.

*(E)-2-((2-(2,4-Dichlorophenyl)hydrazono)methyl)-1-methyl-1H-benzo[d]imidazole* (**6y**): Yellow power, yield: 75.4%, m.p. 211.8–212.9 °C. ^1^H-NMR (400 MHz, DMSO-*d*_6_) δ 10.62 (s, 1H), 8.53 (d, *J* = 1.1 Hz, 1H), 7.67–7.61 (m, 2H), 7.55 (d, *J* = 2.4 Hz, 1H), 7.52 (d, *J* = 8.9 Hz, 1H), 7.39 (dd, *J* = 8.9, 2.4 Hz, 1H), 7.31 (ddd, *J* = 8.2, 7.1, 1.2 Hz, 1H), 7.24 (ddd, *J* = 8.2, 7.2, 1.2 Hz, 1H), 4.13 (s, 3H). MS (ESI): 319.06 (C_15_H_13_Cl_2_N_4_, [M + H]^+^). Anal. Calcd. For C_15_H_12_Cl_2_N_4_: C, 56.44; H, 3.79; N, 17.55. Found: C, 56.31; H, 4.01; N, 17.61.

*(E)-4-(2-((1-Methyl-1H-benzo[d]imidazol-2-yl)methylene)hydrazinyl)benzonitrile* (**6z**): Yellow power, yield: 55.8%, m.p. 258.8–259.0 °C. ^1^H-NMR (400 MHz, DMSO-*d*_6_) δ 11.42 (s, 1H), 8.14 (s, 1H), 7.71 (d, *J* = 8.6 Hz, 2H), 7.63 (t, *J* = 8.3 Hz, 2H), 7.31 (t, *J* = 7.5 Hz, 1H), 7.25 (d, *J* = 7.8 Hz, 1H), 7.19 (d, *J* = 8.5 Hz, 2H), 4.13 (s, 3H). MS (ESI): 329.05 (C_15_H_14_BrN_4_, [M + H]^+^). Anal. Calcd. For C_15_H_13_BrN_4_: C, 54.73; H, 3.98; N, 17.02. Found: C, 54.51; H, 4.01; N, 17.11.

*(E)-1-Methyl-2-((2-(4-(trifluoromethoxy)phenyl)hydrazono)methyl)-1H-benzo[d]imidazole* (**6aa**): Pale yellow power, yield: 68.2%, m.p. 196.8–196.9 °C. ^1^H-NMR (400 MHz, DMSO-*d*_6_) δ 11.07 (s, 1H), 8.07 (s, 1H), 7.61 (t, *J* = 7.9 Hz, 2H), 7.29 (dd, *J* = 11.2, 4.7 Hz, 3H), 7.25–7.20 (m, 1H), 7.16 (d, *J* = 9.0 Hz, 2H), 4.13 (s, 3H). MS (ESI): 335.07 (C_16_H_14_F_3_N_4_O, [M + H]^+^). Anal. Calcd. For C_16_H_13_F_3_N_4_O: C, 57.49; H, 3.92; N, 16.76. Found: C, 57.17; H, 3.89; N, 16.76.

*(E)-2-((2-(4-Fluorophenyl)hydrazono)methyl)-1-methyl-1H-benzo[d]imidazole* (**6ab**): Yellow power, yield: 60.8%, m.p. 183.2–184.1 °C. ^1^H-NMR (400 MHz, DMSO-*d*_6_) δ 10.91 (s, 1H), 8.03 (s, 1H), 7.60 (t, *J* = 8.5 Hz, 2H), 7.27 (dd, *J* = 11.1, 4.1 Hz, 1H), 7.22 (dd, *J* = 11.0, 4.0 Hz, 1H), 7.15(s,1H), 7.14–7.07 (m, 3H), 4.12 (s, 3H). MS (ESI): 269.14 (C_15_H_14_FN_4_, [M + H]^+^). Anal. Calcd. For C_15_H_13_FN_4_: C, 67.15; H, 4.88; N, 20.88. Found: C, 67.23; H, 4.71; N, 20.69.

*(E)-2-((2-(2-Bromophenyl)hydrazono)methyl)-1-methyl-1H-benzo[d]imidazole* (**6ac**): Brown power, yield: 56.6%, m.p. 190.1–190.9 °C. ^1^H-NMR (400 MHz, DMSO-*d*_6_) δ 10.29 (s, 1H), 8.56 (s, 1H), 7.63 (dd, *J* = 14.4, 8.0 Hz, 2H), 7.53 (ddd, *J* = 17.2, 8.1, 1.2 Hz, 2H), 7.37 (t, *J* = 7.3 Hz, 1H), 7.30 (dd, *J* = 11.2, 4.0 Hz, 1H), 7.26–7.20 (m, 1H), 6.87–6.81 (m, 1H), 4.13 (d, *J* = 7.6 Hz, 3H). MS (ESI): 276.13 (C_16_H_14_N_5_, [M + H]^+^). Anal. Calcd. For C_16_H_13_N_5_: C, 69.80; H, 4.76; N, 25.44. Found: C, 69.75; H, 4.74; N, 25.23.

*(E)-6-Methyl-2-((2-phenylhydrazono)methyl)-1H-benzo[d]imidazole* (**6ad**): Yellow power, yield: 64.3%, m.p. 204.6–205.3 °C. ^1^H-NMR (400 MHz, DMSO-*d*_6_) δ 12.40 (s, 1H), 10.87 (s, 1H), 7.84 (s, 1H), 7.48 (t, *J* = 7.1 Hz, 1H), 7.39 (t, *J* = 8.1 Hz, 1H), 7.30 (s, 1H), 7.29–7.25 (m, 2H), 7.25–7.20 (m, 2H), 6.84 (t, *J* = 7.1 Hz, 1H), 2.41 (s, 3H). MS (ESI): 251.10 (C_15_H_15_N_4_, [M + H]^+^). Anal. Calcd. For C_15_H_14_N_4_: C, 71.98; H, 5.64; N, 22.38. Found: C, 71.73; H, 5.31; N, 22.69.

*(E)-2-((2-(4-Chlorophenyl)hydrazono)methyl)-6-methyl-1H-benzo[d]imidazole* (**6ae**): Yellow power, yield: 72.5%, m.p. 216.3–217.5 °C. ^1^H-NMR (400 MHz, DMSO-*d*_6_) δ 12.48 (s, 1H), 11.00 (s, 1H), 7.86 (s, 1H), 7.42 (d, *J* = 8.2 Hz, 1H), 7.34 (s, 1H), 7.31 (s, 2H), 7.24 (d, *J* = 8.7 Hz, 2H), 7.00 (d, *J* = 8.1 Hz, 1H), 2.41 (s, 3H). MS (ESI): 285.09 (C_15_H_13_ClN_4_, [M + H]^+^). Anal. Calcd. For C_15_H_13_ClN_4_: C, 63.27; H, 4.60; N, 19.68. Found: C, 63.11; H, 4.69; N, 19.76.

*(E)-2-((2-(2,4-Difluorophenyl)hydrazono)methyl)-6-methyl-1H-benzo[d]imidazole* (**6af**): Pale yellow power, yield: 59.4%, m.p. 221.9–222.7 °C. ^1^H-NMR (400 MHz, DMSO-*d*_6_) δ 12.51 (s, 1H), 10.81 (s, 1H), 8.13 (s, 1H), 7.80 (td, *J* = 9.3, 5.9 Hz, 1H), 7.46–7.38 (m, 1H), 7.35–7.23 (m, 2H), 7.10 (tt, *J* = 8.8, 1.9 Hz, 1H), 7.01 (d, *J* = 8.2 Hz, 1H), 2.41 (s, 3H). MS (ESI): 287.11 (C_15_H_13_F_2_N_4_, [M + H]^+^). Anal. Calcd. For C_15_H_12_F_2_N_4_: C, 62.93; H, 4.22; N, 19.57. Found: C, 62.83; H, 4.11; N, 19.66.

*(E)-6-Chloro-2-((2-phenylhydrazono)methyl)-1H-benzo[d]imidazole* (**6ag**): Pale yellow power, yield: 72.1%, m.p. 215.6–215.9 °C. ^1^H-NMR (400 MHz, DMSO-*d*_6_) δ 12.73 (s, 1H), 11.03 (s, 1H), 7.87 (s, 1H), 7.63–7.46 (m, 2H), 7.30 (dd, *J* = 8.6, 6.9 Hz, 2H), 7.28–7.23 (m, 2H), 7.21 (dd, *J* = 8.5, 2.1 Hz, 1H), 6.87 (t, *J* = 7.0, 1.4 Hz, 1H). MS (ESI): 271.05 (C_14_H_12_ClN_4_, [M + H]^+^). Anal. Calcd. For C_14_H_11_ClN_4_: C, 62.11; H, 4.10; N, 20.70. Found: C, 61.94; H, 4.19; N, 20.29.

*(E)-6-Chloro-2-((2-(4-chlorophenyl)hydrazono)methyl)-1H-benzo[d]imidazole* (**6ah**): Brown power, yield: 62.8%, m.p. 235.5–236.9 °C. ^1^H-NMR (400 MHz, DMSO-*d*_6_) δ 12.77 (s, 1H), 11.13 (s, 1H), 7.87 (s, 1H), 7.67–7.46 (m, 3H), 7.35 (d, *J* = 2.2 Hz, 1H), 7.34 (d, *J* = 2.3 Hz, 1H), 7.26 (d, *J* = 2.2 Hz, 1H), 7.25 (d, *J* = 2.1 Hz, 1H), 7.21 (d, *J* = 8.8 Hz, 1H). MS (ESI): 305.02 (C_14_H_11_Cl_2_N_4_, [M + H]^+^). Anal. Calcd. For C_14_H_10_Cl_2_N_4_: C, 55.10; H, 3.30; N, 18.36. Found: C, 55.58; H, 3.56; N, 17.88.

*(E)-6-Chloro-2-((2-(2,4-difluorophenyl)hydrazono)methyl)-1H-benzo[d]imidazole* (**6ai**): Yellow power, yield: 68.2%, m.p. 230.2–23.0.7 °C. ^1^H-NMR (400 MHz, DMSO-*d*_6_) δ 12.79 (s, 1H), 10.94 (s, 1H), 8.12 (s, 1H), 7.80 (td, *J* = 9.3, 5.9 Hz, 1H), 7.67–7.58 (m, 1H), 7.49 (d, *J* = 7.9 Hz, 1H), 7.30 (ddd, *J* = 11.8, 8.9, 2.8 Hz, 1H), 7.22 (dd, *J* = 20.5, 8.3 Hz, 1H), 7.12 (tt, *J* = 8.9, 2.1 Hz, 1H). MS (ESI): 306.99 (C_14_H_10_ClF_2_N_4_, [M + H]^+^). Anal. Calcd. For C_14_H_9_ClF_2_N_4_: C, 54.83; H, 2.96; N, 18.27. Found: C, 54.61; H, 3.01; N, 17.98.

### 3.3. Crystallographic Study

X-ray single-crystal diffraction data for compound **6b** were measured on a Bruker SMART APEX CCD area detector diffractometer using MoKα radiation (λ = 0.71073 Å) by the π and ω scan mode. The program SAINT was used for integration of the diffraction profiles. The structure was solved by direct methods using the SHELXS program of the SHELXTL package and refined by full-matrix least-squares methods with SHELXL. All non-hydrogen atoms of compound **6b** were refined with anisotropic thermal parameters. All hydrogen atoms were placed in geometrically idealized positions and constrained to ride on their parent atoms.

### 3.4. Biological Assay

The test fungi *R. solani* and *M. oryzae* were provided by the Laboratory of Plant Disease Control at Nanjing Agricultural University. After removing strains from the storage tube, the strains were incubated in potato dextrose agar (PDA) at 25 °C for a week to develop new mycelia for the antifungal assay.

#### 3.4.1. Antifungal Activity Assays in Vitro

The in vitro antifungal activities of the target compounds against *R. solani* and *M. oryzae* were evaluated using the mycelium growth rate method [19,20]. The compounds were dissolved in DMSO and then mixed with 30 mL sterile molten PDA to obtain final concentrations of 0.625, 1.25, 2.5, 5, 10 and 20 μg/mL (containing 4‰ DMSO) at 50 °C, then PDA with different compounds was poured into 90 mm petri dishes (15 mL/dish), on which 0.5 cm mycelial disks of the two fungi were planted in the center. Each measurement consisted of at least three replicates. After a certain incubation period (1.5 days for *R. solani*, 5 days for *M. oryzae*, according to their different mycelial growth rates) at 25 °C in the dark, mycelia growth diameters were measured. DMSO was served as negative control. The inhibition rate was calculated according to the formula: Inhibition rate = (A − B)/(A − 0.5 cm) × 100%. Where A is the diameter (cm) of the negative control and B is the mycelial diameter (cm) in petri dishes with compounds. Carbendazim, validamycin A and isoprothiolane were co-assayed as the positive control. Those compounds were treated to calculate their median effective concentration (EC_50_) values.

#### 3.4.2. Inhibition of **6f** on the Sclerotia Germination of *R. solani*

Sclerotia of *R. solani* were cultured and dried for germination test [21]. A series of concentrations of tested samples were prepared, then two layers of filter paper were soaked with the solution. These filter paper layers were put in culture dishes and each concentration consisted of at least three replicates. A replicate included 50 sclerotia. Validamycin A and distilled water was served as the positive control and blank control, respectively. These dishes were incubated at 25 °C for 4 days. Then the inhibition rate of the compounds on the germination of sclerotia was calculated as: Inhibition rate = (average germination number of blank control − average germination number of treatment)/average germination number of blank control × 100%.

#### 3.4.3. Protective Activity of **6f** against RSB In Vivo

For evaluating the antifungal activity of **6f** against RSB in vivo, rice cultivar (*Shanyou 63*) was sown and grown in plastic pots in the greenhouse [22]. The compound **6f** at the concentration of 200 μg/mL and 100 μg/mL was sprayed on the cultivar, respectively, and inoculated with *R. solani* 24 h later. Validamycin A at the concentration of 200 μg/mL and 100 μg/mL was used as the positive control. All the treatments were replicated for 20 plants and incubated in the greenhouse with an average midday relative humidity of 85%. A visual disease assessment was made 7 days after inoculating with *R. solani* by measuring the lesion length. The protective efficacy was calculated as: Protection efficacy = ((average lesion length of control − average lesion length of treated group)/average lesion length of control) × 100%.

#### 3.4.4. Inhibition of **6f** on the Conidium Germination of *M. oryzae*

Abundant spores of *M. oryzae* were collected and suspended to a concentration of 5 × 10^4^ spores per milliliter in sterile water for conidium germination test. A series of concentrations of tested samples (**6f** and control) were mixed with conidial suspension, respectively. Aliquots of 10 μL of prepared conidial suspension were placed on separate glass slides in triplicate, which were incubated in a moisture chamber at 25 °C for 24 h. Each slide was then observed under the microscope, and the appressorium formation percentage was examined [23].

#### 3.4.5. Protective Activity of **6f** against RB In Vivo

Compound **6f** was further measured for its antifungal activity in vivo against RB on rice (*Shanyou 63*). Different concentrations (50 μg/mL and 100 μg/mL) of **6f** (4 mL) were sprayed on two-week old seedlings of rice leaves. After 24 h, previously prepared spore suspension of *M. oryzae* was also sprayed with 0.2% (*w*/*v*) gelatin. Plants were kept at 25 °C with 90% humidity and in a 12/12 h light/dark cycle. The positive control is carbendazim of 25 and 50 μg/mL. Lesion formation was observed daily and the protective efficacies were calculated after 7 days [24].

## 4. Conclusions

In conclusion, a series of benzimidazole derivatives had been synthesized and investigated for the antifungal activities against *R. solani* and *M. oryzae* in vitro and in vivo. The result showed that many of the compounds had significant antifungal activities. Compound **6f** exhibited most potent antifungal activity with an EC_50_ value of 1.20 μg/mL against *R. solani*, which was better than carbendazim (1.84 μg/mL). The protective activity of **6f** in vivo reached 66.5% at 100 μg/mL, whereas validamycin A was 61.0%. At the same time, **6f** displayed moderate activity against *M. oryzae* with EC_50_ value of 1.85 μg/mL, and inhibited the conidium germination at 1.0 μg/mL. The result indicated that the compound **6f** with a benzimidazole phenylhydrazone scaffold could be considered as a leading structure for the development of novel fungicides.

## Figures and Tables

**Table 1:** Antifungal activity of compounds against two phytopathogens ^a^.

Compound	R_1_	R_2_	R_3_	EC_50_ (±SD) μg/mL	
				*R. solani*	*M. oryzae*
**6a**	H	H	H	2.86 ± 0.15	11.09 ± 0.92
**6b ^b^**	H	H	2-F	2.14 ± 0.12	6.73 ± 0.56
**6c ^b^**	H	H	3-F	2.14 ± 0.18	9.08 ± 0.63
**6d ^b^**	H	H	4-F	1.88 ± 0.13	8.29 ± 0.28
**6e ^b^**	H	H	4-OCF_3_	1.50 ± 0.08	8.22 ± 0.67
**6f ^b^**	H	H	2,4-2F	1.20 ± 0.10	1.85 ± 0.21
**6g ^b^**	H	H	2,5-2F	3.98 ± 0.19	12.39 ± 0.89
**6h ^b^**	H	H	2-Cl	3.29 ± 0.17	5.47 ± 0.49
**6i ^b^**	H	H	3-Cl	3.77 ± 0.23	13.66 ± 0.19
**6j ^b^**	H	H	4-Cl	1.00 ± 0.06	8.71 ± 0.55
**6k ^b^**	H	H	2,4-2Cl	2.83 ± 0.15	9.27 ± 0.81
**6l ^b^**	H	H	2,5-2Cl	7.47 ± 0.31	14.66 ± 1.17
**6m ^b^**	H	H	2,6-2Cl	5.85 ± 0.22	12.87 ± 0.76
**6n ^b^**	H	H	3,5-2Cl	>25	>25
**6o ^b^**	H	H	2-Br	4.18 ± 0.21	11.33 ± 1.21
**6p ^b^**	H	H	4-Br	1.14 ± 0.07	8.90 ± 1.05
**6q**	H	H	4-OMe	7.02 ± 0.26	16.53 ± 1.24
**6r ^b^**	H	H	4-CN	7.60 ± 0.23	21.18 ± 1.91
**6s ^b^**	H	H	3,4-2CH_3_	6.95 ± 0.29	17.62 ± 1.62
**6t**	H	CH_3_	H	10.04 ± 0.25	>25
**6u ^b^**	H	CH_3_	3-F	>25	>25
**6v ^b^**	H	CH_3_	2,4-2F	2.89 ± 0.04	7.89 ± 0.31
**6w ^b^**	H	CH_3_	2,5-2Cl	>25	>25
**6x**	H	CH_3_	4-Cl	>25	>25
**6y ^b^**	H	CH_3_	2,4-2Cl	>25	>25
**6z**	H	CH_3_	2-Br	>25	>25
**6aa ^b^**	H	CH_3_	4-OCF_3_	>25	>25
**6ab ^b^**	H	CH_3_	4-F	3.01 ± 0.02	>25
**6ac ^b^**	H	CH_3_	4-CN	>25	>25
**6ad ^b^**	CH_3_	H	H	3.84 ± 0.13	19.77 ± 0.65
**6ae ^b^**	CH_3_	H	4-Cl	1.28 ± 0.09	11.85 ± 0.34
**6af ^b^**	CH_3_	H	2,4-2F	2.30 ± 0.11	6.54 ± 0.19
**6ag ^b^**	Cl	H	H	2.65 ± 0.06	14.24 ± 0.58
**6ah ^b^**	Cl	H	4-Cl	0.93 ± 0.04	10.40 ± 0.24
**6ai ^b^**	Cl	H	2,4-2F	1.15 ± 0.09	5.26 ± 0.14
carbendazim				1.84 ± 0.04	1.87 ± 0.10
validamycin A				5.07 ± 0.28	
isoprothiolane					0.02 ± 0.01

^a^ The data of the fungicidal activities were statistically analyzed using Excel to give the results of EC_50_ values. The results were expressed as the mean ± SD of triplicate experiments; ^b^ Compounds that were first reported.

**Table 2:** The inhibition rate of **6f** on the germination of sclerotia of *R. solani* ^a^.

Compound	Treatment (μg/mL)		
	1	10	50
**6f**	0	24.4%	53.1%
validamycin A	26.3%	52.7%	100%

^a^ Values are the average of 3 replicates.

**Table 3:** Protective activity of **6f** against RSB ^a^.

Compound	Treatment (μg/mL)	Lesion Lengtha (mm)	Protection Efficacy (%)
**6f**	200	5.1	68.9
	100	5.5	66.5
validamycin A	200	4.3	73.8
	100	6.4	61.0
blank control		16.4	

^a^ Values are the average of 20 replicates.

**Table 4:** Protective activity of **6f** against RB ^a^.

Compound	Treatment (μg/mL)	Blast Lesions	Protection Efficacy (%)
**6f**	50	32.7	21.6
	100	26	37.6
carbendazim	25	11.3	72.9
	50	5.0	88.0
blank control		41.7	

^a^ Values are the average of 10 replicates.
